# Acid-Base Disorder in the Patients Visiting the Emergency Department of a Tertiary Care Hospital: A Descriptive Cross-sectional Study

**DOI:** 10.31729/jnma.6744

**Published:** 2021-07-31

**Authors:** Sudeep Yadav, Suman Kumar Jha, Neela Sunuwar, Anu Radha Twayana

**Affiliations:** 1 B.P. Koirala Institute of Health Sciences, Dharan, Nepal; 2 Department of High Care Unit and COVID Care Unit, Nidan Hospital Limited, Lalitpur, Nepal; 3 Kathmandu University School of Medical Sciences, Dhulikhel, Nepal

**Keywords:** *acid-base disorder*, *acidosis*, *alkalosis*, *mixed disorder*

## Abstract

**Introduction:**

An acid-base disorder is a change in the normal value of extracellular pH that may result when renal or respiratory function is abnormal or when an acid or base load overwhelms their excretory capacity. Clinical acid-base disorders are conventionally defined from the vantage point of their impact on carbonic-acid-bicarbonate buffer system. The aim of the study is to find out the prevalence of acid-base disorder among patients visiting the emergency department of a tertiary care hospital.

**Methods:**

This is a descriptive cross-sectional study conducted among 370 patients who underwent arterial gas analysis at the emergency department of a tertiary care hospital. The study was carried out from 15th July 2016 to 15th July 2017 after receiving ethical approval from Institutional Review Committee. Convenient sampling was done. Point estimate at 95% Confidence Interval was calculated along with frequency and proportion for binary data. Data were entered in Microsoft-Excel. Statistical Package for Social Sciences version 17 was used for analysis.

**Results:**

Out of 370 patients analyzed, 329 (88.91%) (84.68-91.311 at 95% Confidence Interval) had acid-base disorder. The mixed disorder was the most common finding 80 (21.6%), followed by compensated Respiratory Acidosis 56 (17.8%). The mean age group of male patients studied was 50.72±20.586. and among females, it was 49.95±20.908. Among those most common symptoms were shortness of breath 151 (40.81%) followed by vomiting 91 (24.59%).

**Conclusions:**

Most common acid-base disorder was mixed disorder presenting with prominent symptoms of shortness of breathe in non-geriatric patients wherein the geriatric patient, the most common disorder was compensated respiratory acidosis with the prominent symptom of shortness of breath.

## INTRODUCTION

An acid base disorder is a change in the normal value of extracellular pH that occurs when renal or respiratory function is abnormal or when an acid or base loads overwhelms their excretory capacity.^1^

An arterial blood gas (ABG) measures the arterial oxygen tension (PaO2), carbon dioxide tension (PaCO2), acidity (pH) and bicarbonate level (HCO3-). It helps to determine the effectiveness of oxygen therapy. It is helpful in diagnosis and management of the patients who may also require intensive care facilities. This study contributes towards formation of guidelines while dealing with such patients requiring critical care.

The primary objective of this study is to find out common acid base disorder in the patients. The secondary objectives are to evaluate the common causes of acid-base disorder, find the occurrence of prominent symptoms in different acid-base disorder, and to find out the association of acid-base disorder with age and sex if any.

## METHODS

This is a descriptive cross-sectional study done on patients visiting BPKIHS for ABG analysis. Following approval from the Institutional Review Committee of B.P. Koirala Institue of Health Sciences. All the patients who underwent ABG at the emergency department laboratory of BPKIHS from 15th July, 2016 to 15th July, 2017 were taken. Waiver of the requirement for informed consent was obtained under Institutional Review Committee regulation. We included patients who had done ABG at emergency department laboratory (only first sample from a patient). Patients who were admitted to ICU were excluded. No more than one sample from same patient was included in the study. Convenient sampling was done.

The sample size was calculated as below:

n = Z^2^ × p × q / e^2^

  = (1.96)^2^ × (0.5) × (0.5) / (0.06)^2^

  = 264

Where,

n = required sample size
Z = 1.96 for 95% Confidence Interval
p = prevalence of acid base disorder, 50%
q = 1-p
e = margin of error, 6%

The minimum sample size calculated was 264. A total sample of 370 was taken for the study. The records of the ABG of the patients in the emergency department were reviewed and data regarding the ABG parameters were collected and recorded in a preset proforma. Similarly, patients information, chief complaint, age, sex, diagnosis and others were also included from Medical-Record section through patients' files.

The data were entered and cross-checked in Microsoft Excel. Acid-Base disorder was interpreted manually for each data and cross-checked by using android software complete ABG. The results were expressed as mean+/-SD for quantitative data and percentage for the qualitative data. Statistical Package for Social Sciences version 17.0 was used for the analysis.

## RESULTS

Out of 370 patients analyzed, 329 (88.91%) (84.68-91.311 at 95% Confidence Interval) had acid base disorder. Mixed disorder was the most common finding 80 (21.6%), followed by compensated respiratory acidosis 56 (17.8%) (Table 1).

There were 187 (50.5%) males and 183 (49.45%) females, within the age group of 1 to 99 years. Mean age group of male patients studied was 50.72±20.586 and among females it was 49.95±20.908.

**Table 1:** Frequency and percentage of Acid-base disorders.

Acid-Base Disorders	n (%)
Normal	41 (11.1)
Mixed Disorder	80 (21.6)
Compensated Respiratory Alkalosis	58 (15.7)
Compensated Respiratory Acidosis	66 (17.8)
Compensated Metabolic Alkalosis	52 (14.1)
Compensated Metabolic Acidosis	39 (10.5)
Respiratory Alkalosis	3 (0.8)
Respiratory Acidosis	3 (0.8)
Metabolic Alkalosis	6 (1.6)
Metabolic Acidosis	22 (5.9)
Total	370 (100)

Out of 370 patients, following diagnosis were identfiiec (Table 2).

**Table 2:** Frequency of Diseases.

Diagnosis	n (%)
Cardiac Failure	15 (4.1)
CKD	18 (4.9)
COPD	39 (10.5)
CVA	8 (2.2)
Diabetes Mellitus(DM)	23 (6.2)
Hypovolemic shock	38 (10.3)
Infectious Disease	43 (11.6)
Liver Disease	16 (4.3)
Poisoning	45 (12.2)
Other Respiratory Disease^*^	55 (14.9)
Others^**^	70 (18.9)
Total	370 (100)

* Pneumonia, Tuberculosis, Pleuraleffusion, Asthma,Carcinoma of lung, Interstitial Lung Disease.

** Non-specific disease, Hypertension, Surgical conditions, Myocardial Infarction, Obstetrics & Gynaecological diseases.

The frequent presenting symptoms were Shortness of Breath (SOB) 151 (40.81%) followed by vomiting 91 (24.59%).

About 370 cases were divided in two categories by age according to senior citizen act 2063 B.S Nepal; geriatric (>60yr) and non-geriatric (<60yr). The most common disorder among non-geriatric population was mixed disorder 50 (22.22%) followed by compensated respiratory alkalosis 37 (16.44%) and among geriatric was compensated respiratory acidosis 32 (22.07%) followed by mixed disorder 30 (20.69%) as shown in (Table 3).

**Table 3:** Acid-base disorders in <60 years and >60 years

Disorder	Non Geriatric (<60yr) n (%)	Geriatric (>60yr) n (%)
Normal	28 (12.44)	13 (8.96)
Mixed Disorder	50 (22.22)	30 (20.69)
Compensated Respiratory Alkalosis	37 (16.44)	21 (14.48)
Compensated Respiratory Acidosis	34 (15.11)	32 (22.07)
Compensated Metabolic Alkalosis	24 (10.67)	28 (19.31)
Compensated Metabolic Acidosis	28 (12.44)	11 (7.59)
Respiratory Alkalosis	1 (0.44)	2 (1.38)
Respiratory Acidosis	2 (0.89)	1 (0.69)
Metabolic Alkalosis	4 (1.78)	2 (1.38)
Metabolic Acidosis	17 (7.56)	5 (3.45)
Total	225 (60.81)	145 (53.70)

The relation between frequency of acid-base disorder with sex is shown in (Table 4).

**Table 4:** Frequency of Acid-Base Disorders in relation to sex.

Disorder	Female n (%)	Male n (%)
Normal	22 (5.94)	22 (5.13)
Mixed Disorder	40 (10.81)	40 (10.81)
Compensated Respiratory Alkalosis	28 (7.56)	28 (8.10)
Compensated Respiratory Acidosis	32 (8.64)	32 (9.18)
Compensated Metabolic Alkalosis	26 (7.02)	26 (7.02)
Compensated Metabolic Acidosis	19 (5.13)	19 (5.40)
Respiratory Alkalosis	2 (0.54)	2 (0.27)
Respiratory Acidosis	2 (0.54)	2 (0.27)
Metabolic Alkalosis	1 (0.27)	1 (1.351)
Metabolic Acidosis	11 (2.9)	11 (2.97)
Total	183 (49.45)	183 (50.54)

## DISCUSSION

Our study revealed Mixed disorder (21.6%) is most common acid-base disorder followed by compensated respiratory acidosis (17.8%) as shown in Table 1. This observation is consistent with Anderson, et al. whose prospective survey of over thousand consecutive ABG samples obtained from patient in intensive medical care unit showed that 51% of patient had mixed acid-base disorders and Shivshankar, et al. whose descriptive study over 100 cases showed that 72% had a mixed disorder.^2,3^

The most common causes of Acid-base disorders are others (18.9%), other respiratory disease (14.9%), poisoning (12.2%), and infectious (11.6%) as shown in Table 2. In COPD patients, the most common acid-base disorder was compensated respiratory acidosis (35.89%) followed by mixed disorder (23.07%) as supported by review article by Cosimo M B and Maria V about Acid-base disorder in patients with COPD.^4^ Compensated respiratory acidosis is explained by experimental studies which show that total NHE3 and NBCe1 protein abundance are upregulated by chronic respiratory acidosis.^5^ However, the main mechanism responsible for the elevation in serum bicarbonate is the increased excretion of titratable acid and ammonium, which are stimulated by persistently elevated pCO_2._^6^

The generalized diagnosis of DM in this study includes Diabetic Ketoacidosis, diabetic foot, DM with superimposed infections. This might be the probable reason that our study showed 30.43% of mixed disorder, 26.08% of compensated metabolic alkalosis,and 13.04% of compensated metabolic acidosis as shown in Table 3. This study is supported by Shivshankar S, et al. article which showed that mixed acid base disorders were more common in DKA than simple acidosis and compensated metabolic alkalosis due to use of diuretics.^3^

In cases of poisoning, result shows 33.33% had compensated respiratory acidosis, compensated metabolic acidosis (15.55%), and mixed disorder (13.33%) as in Table 3. Respiratory acidosis might be due to CNS depression or airway obstruction by poison leading to hyperventilation.

In case of infectious disease, mixed disorder (25.58%) and compensated respiratory alkalosis (25.58%) are more common as shown in Table 3. This might be due to endogenous stimulation of respiratory centre causing respiratory alkalosis, lactic acidosis causing metabolic acidosis and respiratory failure causing respiratory acidosis.^3^

In case of cardiac failure, result shows compensated metabolic alkalosis (33.33%), compensated respiratory alkalosis (26.67%) are common as shown in Table 3. Diuretics employed in heart failure are frequently responsible for metabolic alkalosis due to several possible mechanisms. Diuretics cause an increase in sodium delivery to the distal nephron and accelerate potassium and proton secretion; furthermore, volume contraction stimulates renin and aldosterone secretion. Potassium depletion with an aldosterone excess is always accompanied by metabolic alkalosis. In CHF, various acid-base disorders can be discovered due to the renal loss of hydrogen ions and hydrogen ion movements into cells, the reduction in the effective circulating volume, hypoxemia, and renal failure. This justifies the occurrence of metabolic alkalosis, metabolic acidosis, respiratory alkalosis, as well as respiratory acidosis alone or in combination.^7^

In cases of CKD, the most common acid-base disorders are compensated metabolic acidosis (33.33%) followed by metabolic acidosis (27.78%) as shown in Table 3. The major reason for metabolic acidosis in CKD is due to decrease in total ammoniagenesis as a result of decreasing numbers of functioning nephrons.^8^

In case of CVA, our result shows compensated respiratory alkalosis (25%), probably due to central hyperventilation as suggested by Shivshankar S, et al. article.^3,9^

In case of liver diseases, most common disorder is compensated respiratory alkalosis (25%) followed by compensated metabolic acidosis (18.75%) and mixed disorder (18.75%) as shown in Table 3, probably because hyperventilation is an almost universal finding with advanced liver disease, leading to chronic respiratory alkalosis.^10^

In overall disorders, the most frequently presented symptoms are SOB (40.81%) followed by vomiting (24.59%). Among mixed disorder, the most common presenting symptom is SOB (37.5%) followed by other non-specific symptoms (28.75%). In compensated respiratory acidosis, SOB (46.96%) followed by vomiting (30.3%) are prominent presenting symptoms. In compensated respiratory alkalosis, the most common presenting symptom is SOB (36.40%) as shown in Table 4.

Among non-geriatric (<60yr) patients, the most common acid-base disorder is mixed disorder (22.22%) followed by compensated respiratory alkalosis (16.44%).

Among geriatric (>60yr) patients, compensated respiratory acidosis (22.07%) is the most common disorder followed by mixed disorder (20.69%) as shown in Table 5.

In our study, no any relation has been found between sex and acid-base disorders as shown in Table 6.

In our result, lactate level is high in liver disease (2.5±3.39SD) and hypovolemic shock (2.31±2.02SD) as shown in Table 11. Increased lactate level in liver disease is due to combination of accelerated glycolysis in the splanchnic region and a reduction in hepatic gluconeogenesis.^8^ In case of hypovolemic shock, it is due to tissue hypoxia.

The study shows that the most common electrolyte imbalance in liver disease is hyponatremia along with normokalemia. Similar results had been observed in the study done by Jong Hoon Kim, et al. Shubhada N and Ahya, et al. probably due to impaired free water clearance.^6,10^

In COPD, hyponatremia with hypokalaemia is most common electrolyte imbalance observed in our study which is also suggested by G Valli, et al.^8^

In our study most common electrolyte imbalance is hyponatremia among all other diagnosis.

Although it is critical to evaluate the blood oximetry in managing patient in emergency set up, the evaluation of other blood parameters such as electrolytes, glucose, lactate, osmolarity etc often remains neglected. These parameters will provide additional information on the patients ABG status, relating the cause to affect phenomenon. In addition, the diagnostic efficiency will be enhanced by minimizing the cost and time. This would have been a problem if these tests had to be requisitioned as separate test or in serum as there is not much difference in the values of these parameters in venous and arterial blood.

## CONCLUSIONS

The most common acid-base disorder was mixed disorder presenting with shortness of breath as the most prominent symptom in non-geriatric patients but in geriatric patients, the most common disorder was compensated respiratory acidosis with shortness of breath being the most prominent.
